# A haplome alignment and reference sequence of the highly polymorphic *Ciona savignyi *genome

**DOI:** 10.1186/gb-2007-8-3-r41

**Published:** 2007-03-20

**Authors:** Kerrin S Small, Michael Brudno, Matthew M Hill, Arend Sidow

**Affiliations:** 1 Departments of Pathology and of Genetics, Stanford University Medical Center, 300 Pasteur Drive, Stanford, California 94305-5324, USA; 2 Department of Computer Science, Banting and Best Department of Medical Research, University of Toronto, Toronto, 6 King's College Rd, Ontario, M5S 3G4, Canada

## Abstract

The sequence of *Ciona savignyi *was determined using a whole-genome shotgun strategy, but a high degree of polymorphism resulted in a fractured assembly wherein allelic sequences from the same genomic region assembled separately. We designed a multistep strategy to generate a nonredundant reference sequence from the original assembly by reconstructing and aligning the two 'haplomes' (haploid genomes). In the resultant 174 megabase reference sequence, each locus is represented once, misassemblies are corrected, and contiguity and continuity are dramatically improved.

## Abstract 2

The high degree of polymorphism in the genome of the sea squirt *Ciona savignyi *complicated the assembly of sequence contigs, but a new alignment method results in a much improved sequence.

## Background

We describe the generation of the reference sequence for the *Ciona savignyi *genome. *C. savignyi *is among the species of sessile marine invertebrates commonly known as sea squirts. It is an important model organism [1] that is amenable to a variety of molecular genetic experiments [2]. As a urochordate, it occupies an advantageous phylogenetic position, sharing conserved developmental mechanisms with vertebrates while being a substantially simpler organism both genomically and developmentally [3,4]. In addition, a draft genome sequence of a sister *Ciona *sp. (*C. intestinalis*) [5] is available, further enhancing the experimental and comparative value of a high-quality *C. savignyi *genome sequence [6,7].

The *C. savignyi *genome project employed a whole-genome shotgun (WGS) strategy to sequence a single, wild-collected individual to a depth of 12.7× [8]. Assembly was complicated by an unexpected and extreme degree of heterozygosity [8], because current WGS assembly algorithms (including Arachne [9], the assembler employed for this genome) are not designed to accommodate highly polymorphic shotgun data [9-13]. The best shotgun assemblies have thus far been produced from species that exhibit a low rate of polymorphism (for instance, human [14]) or from inbred laboratory or agricultural strains (for example, mouse [15], *Drosophila melanogaster *[16], and chicken [17]). Assemblies of genomes from organisms with moderate or localized heterozygosity encountered significant difficulty that resulted in lower quality than expected, given the depth of sequencing [5,18-21].

An alternate WGS assembly strategy was developed for the *C. savignyi *genome that leveraged the extreme heterozygosity and depth of the shotgun data to force separate assembly of the two alleles [8] across the entire genome. In the resulting WGS assembly, nearly all loci are therefore represented exactly twice. However, the assembler had no mechanism by which to determine which contigs were allelic. Thus, the redundant WGS assembly contains no information to indicate how the constituent contigs relate to the two 'haplomes' (haploid genomes), preventing selection of a single haplome as a reference sequence. Importantly, there is also no distinction between highly similar contigs that represent two different alleles and those resulting from paralogous regions.

The redundancy of the original WGS assembly represented a practically insurmountable problem for genome annotation. Available genome data structures and browsers require a nonredundant reference sequence, and current gene prediction pipelines are highly parameterized and dependent on a hierarchy of heuristics that cannot accommodate the presence of two alleles in a single assembly [22-24]. Additionally, if a redundant gene set were to be obtained, then the lack of distinction between alleles and paralogs would significantly complicate evolutionary analyses, which are among the primary uses of the *C. savignyi *genome.

It was therefore imperative to generate a reference sequence for *C. savignyi *that could serve as a nonredundant resource and as the basis for genome annotation. We here describe how we generated the nonredundant, high-quality reference sequence, using the original WGS assembly as a starting point. Our strategy first identified allelic contigs and supercontigs in order to reconstruct the two haplomes and enable construction of a pair-wise haplome alignment. The aligned haplomes were then utilized to identify and, where possible, to correct several types of misassembly. The alignment also allowed the bridging of contig and supercontig gaps in one haplome by the other, dramatically improving long-range contiguity. Finally, the alignment was parsed to generate a composite nonredundant reference sequence that is more complete than either haplome.

## Results

### Generation of the reference sequence

**Figure 1:**
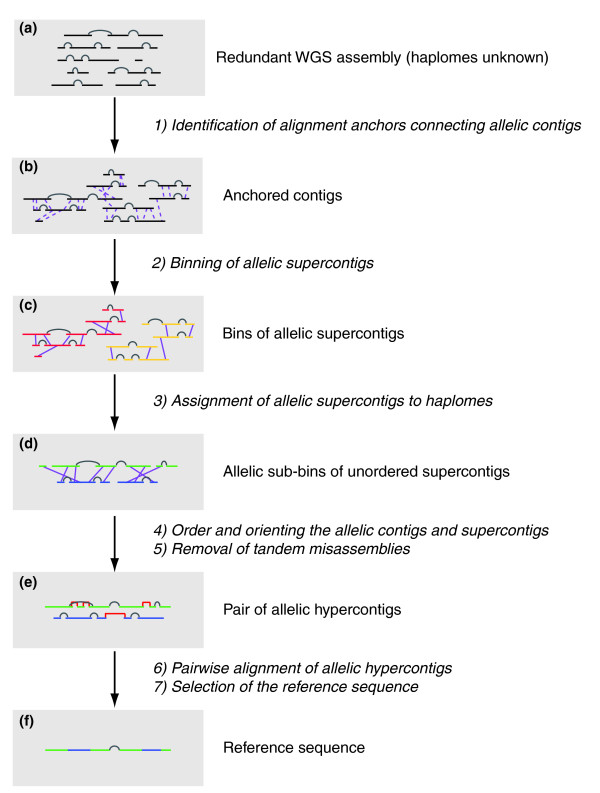
Overview of generation of the *Ciona savignyi *reference sequence. **(a) **The initial whole-genome shotgun (WGS) assembly is represented; black horizontal lines represent contigs, which are connected into supercontigs by gray arcs. **(b) **Dashed purple lines represent unique anchor between allelic contigs. **(c) **Two separate bins are represented by red and yellow supercontigs. **(d) **A single bin is represented; supercontigs in the bin have been assigned to sub-bin A (green) or B (blue). Purple lines denote alignments between allelic contigs in sub-bins A and B. **(e) **An allelic pair of ordered hypercontigs is represented. Red brackets denote regions where alignment to the opposite allele has bridged a supercontig boundary. **(f) **The reference sequence contains sequence from allele A (green) and allele B (blue).

We designed a semiautomated alignment pipeline to generate a nonredundant reference sequence from the original, redundant WGS assembly (Figure 1). The pipeline is comprised of several stages and incorporates purpose-built and existing algorithms. A fully automated pipeline was not attempted because the complexity of the polymorphic assembly required manual inspection at several stages. Our strategy is best described as consisting of seven stages: identification of alignment anchors connecting allelic contigs; binning of allelic supercontigs; assignment of allelic supercontigs to haplomes; ordering and orienting the allelic contigs and supercontigs; removal of tandem misassemblies; pair-wise alignment of allelic hypercontigs; and selection of the reference sequence.

#### Stage 1: identification of alignment anchors connecting allelic contigs

Like all WGS assemblies, the original WGS assembly of *C. savignyi *consists of a set of supercontigs that are comprised of ordered and oriented contigs (Figure 1a). Contigs are connected into supercontigs by paired sequence reads, which are obtained from opposite ends of a single clone. The original WGS assembly contains two copies of most loci, but individual contigs contain no information to indicate which of the two haplomes they belong to or any information to identify allelic contigs.

To identify high confidence allelic regions for use as anchors in later alignment steps, the original WGS contig assembly was soft-masked with a *C. savignyi de novo *RECON [25] repeat library and aligned to itself via a stringent optimization of blastn [26]. Regions of at least 100 consecutive base pairs with exactly one high-quality blast hit were selected as allelic anchors. The requirement for exactly one hit precludes anchors between low copy repeats or duplicated regions. Anchors were filtered to remove those that lie in predominantly masked regions and between contigs in the same supercontig. (As is discussed below, a common error in WGS assembly of polymorphic genomes is tandem misassembly of alleles into the same supercontig; the 6,864 within-supercontig anchors most likely represent instances of this error.) After the filtering step, 239,635 anchors connecting 28,930 contigs remained (Figure 1b).

In order to weight anchors for later steps, a LAGAN [27] global alignment was generated for each anchored contig pair, and a modified alignment score was calculated from each such alignment. The anchored contig pairs and their alignment scores were then mapped to supercontigs. A total of 3,678 supercontigs, comprising 88% of bases in the assembly, contained at least one anchor to another supercontig (Table 1). Of a total 6,411 anchored supercontig pairs, 4,546 were connected by a single contig pair, 723 by exactly two contig pairs, and the remaining 1,142 were connected by more than two contig pairs.

**Table 1:** Sequence in the alignment pipeline

	Number of supercontigs	Number of contigs	% of original sequence
Original WGS assembly	33,623	66,800	100%
Original assembly >3 kb	4,123	37,300	92%
Anchored supercontigs	3,678	34,568	88%
Binned supercontigs	2,360	32,641	85%
Reference sequence	374	3,576	N/A

kb, kilobases; WGS, whole-genome shotgun.

#### Stage 2: binning allelic supercontigs

**Figure 2:**
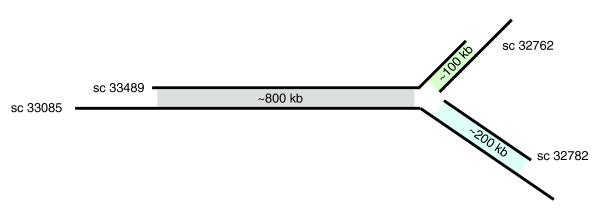
A spatially inconsistent set of alignments ('spider'). Black lines represent aligned supercontigs. Shaded regions between supercontigs correspond to alignments between supercontigs. This alignment conflict is indicative of a major misassembly (Figure 3a) in either supercontig 33,489 or 33,085. Genetic mapping revealed supercontig 33,489 to contain the misassembly, which was corrected by manually breaking it, retaining supercontigs 33,085 and 32,782, and the portion of supercontig 33,489 aligned to 33,085 (shaded gray) together, and placing supercontig 32,762 and the region of 33,489 aligned to 32,762 (shaded green) into a separate bin.

The anchored supercontigs were then sorted into 'bins', defined as collections of supercontigs containing both alleles of a region (Figure 1c) that have no assembly connections to their neighboring regions, as follows. Anchored supercontig pairs were ranked by the sum of their contig-contig LAGAN alignment scores and iteratively grouped starting with the highest ranked pair. Summing the contig LAGAN alignment scores across supercontigs and ranking supercontig pairs in order of scores in effect creates a voting scheme, wherein a spurious alignment or a small paralogous region will be outvoted by the correct allelic alignments of the surrounding sequence. Lower ranking alignments were flagged if they were not spatially consistent with a higher ranking alignment. For example, in Figure 2 the alignment shown in green would be flagged because it creates a linear inconsistency with the higher ranking alignment shown in blue. A total of 2,360 supercontigs comprising 85% of the original WGS assembly were thus sorted into 374 bins (Table 1). A total of 1,318 supercontigs, representing 3% of bases in the original WGS assembly, contained anchors that were overruled during the binning process, and were therefore not assigned to a bin.

**Figure 3:**
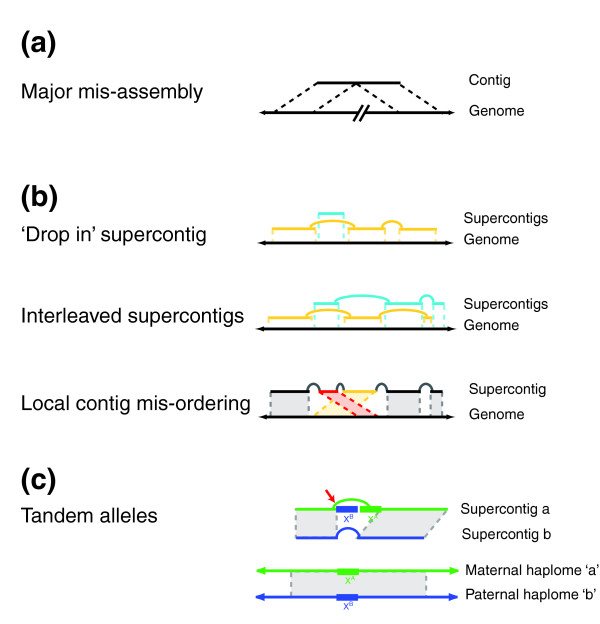
Types of identified misassemblies. In A and B, black arrows correspond to the actual genome, and other lines to the assembly. **(a) **Major misassembly, wherein a single contig (or supercontig) contains sequence from disparate regions of the genome. **(b) **Three types of misassembly that can be corrected by reordering of contigs. Distinct supercontigs are colored yellow or turquoise. **(c) **Allelic regions are placed in tandem (top), instead of correctly into their respective haplomes (bottom). Haplome A sequence is shown in green and haplome B in blue. A sequence misjoin at the location indicated by the red arrow places the X region of haplome B into a haplome A contig. The haplome B supercontig contains an assembly gap in the X region.

Visual inspection of all bins indicated that the majority of the flagged, spatially inconsistent alignments were indeed spurious, but it also revealed loci where the independently assembled allelic supercontigs have a disagreement in long range contiguity, and hence are indicative of a major misassembly in one supercontig (Figures 2 and 3a). A major misassembly occurs when two distinct regions of the genome are joined (usually in a repeat), creating an artificial translocation event [9-13]. Major misassemblies are relatively rare but they are known to occur in nearly all established WGS assemblers and are extremely difficult to detect without a finished sequence or physical map [28-31]. We identified 13 alignment conflicts that were indicative of a major misassembly, and that linked 22 bins into eight 'spiders', so-called because of the branching structure created by the misassembly (Figure 2).

To determine which of the conflicting supercontigs contained the misassembly, we placed genetic markers at relevant locations surrounding each alignment conflict and typed them in a mapping panel. Markers bridging a major misassembly should have no significant linkage or a genetic distance grossly out of scale to the physical distance indicated by the supercontig assembly. In six of the 13 alignment conflicts linkage analysis indicated a clear relationship between markers spanning the putative major misassembly in only one of the supercontigs, and we broke the opposite supercontig sequence accordingly. A detailed representative example is shown in Additional data file 1. In three conflicts we could not locate appropriate fully informative markers and in four conflicts the linkage data were inconclusive. In these cases we parsimoniously broke one supercontig. We note that, given the extreme polymorphism of *C. savignyi*, it is possible that the inconclusive linkage data reflect a polymorphic rearrangement event segregating in the population, because the individuals from the genetic cross were unrelated to the sequenced individual.

**Figure 4:**
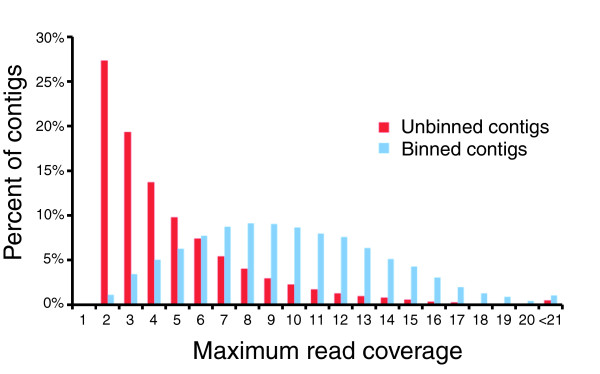
Unassigned contigs are heavily enriched for low sequence coverage. The x-axis is the maximum read coverage per contig, and y-axis is the percentage of contigs in a category. Red bars are unassigned contigs, and blue bars are contigs assigned to an allelic bin.

In total, supercontigs constituting 15% of the bases in the original WGS assembly lacked a unique anchor or were unplaced during the binning process, and were therefore not included in subsequent steps. We suspect that most of the unassigned sequence is comprised of uncondensed repetitive regions and hence, if fully assembled, would represent significantly less than 15% of the genome. This view is supported by several lines of independent evidence. First, 75% of the bases in the unassigned sequence are repeat masked. This is a significant enrichment compared with the original WGS assembly, in which 30% of bases are repeat masked. Second, the unassigned sequence primarily consists of short single-contig supercontigs, whose N50 is only 6 kilobases (kb). Most importantly, the unassigned contigs exhibit a striking preponderance of low sequence coverage: 27% of unassigned contigs have a maximum read coverage of two, whereas only 1.2% of the contigs that were assigned to a bin fall into this category (Figure 4). The mean read coverage per position in the unassigned sequence is 3.7, which is well below the mean of binned contigs of 5.3 (Additional data file 2).

#### Stage 3: assignment of allelic supercontigs to haplomes

Supercontigs in each bin were assigned to one or the other of the two haplomes by leveraging the alignment connections between supercontigs within each bin to assign supercontigs into allelic sub-bins 'A' and 'B' (Figure 1d). A bipartite graph was constructed for each bin, where nodes are supercontigs and edges are alignments between them. We arbitrarily assigned one node of the most trustworthy edge (as determined by alignment score) to sub-bin A. All nodes connected to the initial node by alignment were assigned to sub-bin B. We then traversed the graph one edge at a time and assigned each unassigned node to the opposite sub-bin of the previous node. As the designation of A and B is arbitrary within each bin, the reconstructed haplomes are necessarily a mosaic of the parental contributions.

#### Stage 4: ordering and orienting the allelic contigs and supercontigs

We utilized a purpose-built Java tool to inspect bins for inconsistencies between order/orientation of contigs as suggested by the original WGS assembly and that suggested by the alignments between allelic contigs. The Java graphical user interface displayed all contigs in each bin, their alignment anchors to the other allele, paired reads between distinct supercontigs, and other pertinent information. Manual inspection of all bins revealed the vast majority of inconsistencies to be due to obviously spurious alignments. However, it became clear at this point that it would not be sufficient to chain supercontigs linearly in each sub-bin to obtain the correct order and orientation in each of the two haplomes, because a substantial number of clearly correct alignments between allelic contigs did not conform to the supercontig-imposed ordering, indicating the presence of misassemblies that should be corrected.

Most disagreements could be classified into three types of misassemblies: 'drop-in' supercontigs, interleaved supercontigs, and local contig misorderings (Figure 3b). The most frequent type was of the drop-in variety, in which short, usually single-contig supercontigs were ordered by the alignment to a position entirely within a gap of a different supercontig (Figure 3b). The size of the 'drop-in' supercontig and the gap length of the supercontig into which it would be embedded (as estimated by Arachne) were often strikingly similar, and in many cases the existence of multiple, consistent paired reads between the small supercontig and the encompassing supercontig further supported the alignment-ordered placement. Interleaved supercontigs, which are characterized by the alignment-directed ordering of the terminal contig(s) of one supercontig within a supercontig gap in the adjacent supercontig, were less frequent (Figure 3b). Interleaved supercontig misassemblies have been observed in other WGS assemblies [29]. The final type of misassembly detected at this stage of the pipeline, namely local contig misordering, consists of incorrectly ordered contigs within a single supercontig (Figure 3b), and has been reported in assemblies by all major WGS assemblers [9-13,29].

We developed an algorithm, called the Double Draft Aligner (DDA) [32], to order contigs automatically within each sub-bin with respect to their allelic contigs from the other sub-bin. The DDA operates on contigs rather than supercontigs to allow for the reordering of contigs that would be necessary to correct the three types of misassembly described above (Figure 3b). The DDA is similar to SLAGAN [33], with the notable exception that there is no reference sequence according to which the other input sequence is rearranged. Instead, each of the two input sequences is a set of unordered contigs and either sequence may contain a rearrangement. The DDA constructs 'alignment links' from local alignments between contigs of opposite sub-bins, and utilizes these alignment links to chain contigs iteratively within each of the two sub-bins. In the absence of an alignment link, contigs are chained according to the order within their supercontig. A detailed description of the DDA algorithm is provided in Materials and methods (below). The DDA does not chain across 'unreliable' contigs (contigs with multiple, inconsistent alignments) and a final, manual proofreading step using the Java tool mentioned above was used to correct these cases.

**Figure 5:**
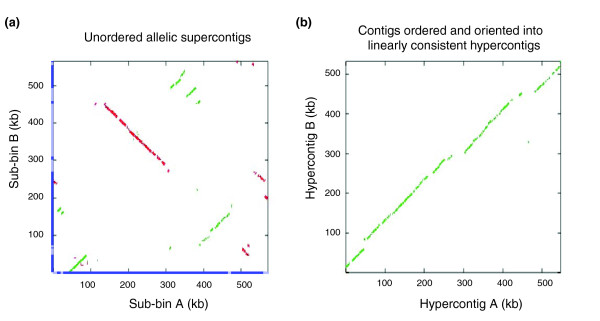
Dotplots of sequence similarity in an allelic bin before and after ordering into hypercontigs by DDA. The x-axis and y-axis in both plots represent sequence from sub-bins A and B, respectively, and cover approximately 550 kilobases (kb). In both plots green dots record a region of sequence similarity on the positive strand and red dots sequence similarity on the negative strand. **(a) **Before the Double Draft Aligner (DDA) is run on this bin, supercontigs from each sub-bin are unordered and not oriented with respect to one another; their locations are denoted by alternating light and dark blue lines along the appropriate axis. **(b) **After the DDA is run, contigs from both sub-bins have been ordered and oriented to produce a pair of linearly consistent hypercontigs.

The effectiveness of the DDA is illustrated in Figure 5. Before the DDA is run on a bin, supercontigs from each sub-bin are unordered. After the DDA is run, the ordering of the contigs in each allele corresponds linearly to the other allele. Once ordered by the DDA, the contigs of each sub-bin were concatenated into a single 'hypercontig' (Figure 1e). The allelic hypercontigs constitute the reconstructed haplome assembly.

It should be noted that some differences in contig order between the haplomes are probably the result of a polymorphic rearrangement rather than a misassembly. The DDA will force contigs of a polymorphic rearrangement to correspond to the order of the more contiguous haplome, and hence introduce an artificial rearrangement in the other haplome. However, because both orderings were present in the sequenced individual and we have no information to indicate which is 'wild type', either ordering is equally legitimate for our primary goal of selecting a nonredundant reference sequence. This also applies to polymorphic inversions, which the DDA identifies but does not re-orient. All identified inversions that spanned at least one entire contig were manually inspected and re-oriented in one hypercontig.

#### Stage 5: removal of tandem misassemblies

The ordered and oriented contigs allowed identification of tandem allele misassemblies, which are known errors of polymorphic WGS assemblies [34]. In a tandem allele misassembly, two alleles of a region are positioned adjacent to each other in the same supercontig (Figure 3c). The insertion of the second, misassembled allele into the supercontig creates a disparity between the length of the assembled sequence and the estimated distance to adjacent contigs provided by paired reads, because the paired reads will 'leapfrog' the misassembled allele (Figure 3c). The assembler is then forced to report a contig overlap to reconcile the conflicting sequence and paired read data. Tandem allele misassemblies are probably common in the original WGS assembly, because the assembler predicted a contig overlap between 5.3% of all adjacent contigs. The predicted overlaps have a total length of 9 megabases (Mb), a median length of 3.7 kb, and an N50 length of 6.4 kb (Additional data file 3). Manual inspection of a sampling of predicted overlaps revealed a strong enrichment for paired read structures, which is indicative of tandem misassembly. An additional 36% of adjacent contigs in the original WGS assembly are predicted to have a gap of length zero, which, given the inherent error in estimating insert lengths, may also include a substantial number of overlapping contigs.

We designed a tool to identify and remove tandem allele misassemblies in adjacent contigs on the basis of alignments within an allelic bin (see Materials and methods, below). A tandem misassembly was identified and removed in 26% of contigs for which the assembler had predicted an overlap, in 5% of contigs with a predicted gap of length zero, and in only 1% of contigs that had a predicted gap of positive length (Table 2). The mean and median length of tandem allele misassemblies was significantly shorter in adjacent contigs with a predicted gap of length zero, as would be expected. In addition, contig overlaps were identified and removed in 11% of adjacent contigs for which no gap estimate was available. This includes terminal contigs in adjacent supercontigs and contigs rearranged by the DDA. Overlapping regions in this category tended to be shorter and nearly identical; these most likely represent sequence from the same allele that should have been merged in the shotgun assembly, rather than a tandem misassembly.

**Table 2:** Identification and removal of tandemly misassembled alleles

	Predicted contig overlap (*n *= 1,496)	Predicted gap of length zero (*n *= 12,434)	Predicted gap of length one or more (*n *= 16,747)	No estimate: rearranged contigs or adjacent supercontigs (*n *= 4,124)
Tandem instances	395 (26%)	581 (5%)	148 (1%)	436 (11%)
Total sequence removed (kb)	1994	888	412	904
Median length (kb)	3.5	0.8	1.4	0.7
Mean length (kb)	5.0	1.6	2.8	2.0

kb, kilobases.

#### Stage 6: pair-wise alignment of allelic hypercontigs

The two reconstructed haplomes consist of 374 pairs of allelic hypercontigs, which contain a total of 336 Mb, including 13 Mb of gaps. Each pair of allelic hypercontigs was globally aligned with LAGAN to produce the final whole genome alignment of the haplomes. The total alignment length is 214 Mb, of which 118 Mb are aligned positions, 47 Mb are gapped positions corresponding to haplome-specific sequence (polymorphic insertion/deletion events such as those resulting from mobile element activity), and 47 Mb (38 Mb of sequence plus 9 Mb of supercontig gap placeholders) of sequence aligned to an assembly break in the opposite hypercontig. The haplome alignments are available on the Sidow laboratory website [35].

#### Stage 7: selection of the reference sequence

A nonredundant reference 'reftig' combining sequence from both haplomes was parsed directly from each hypercontig alignment (Figure 1f). Reftigs were constructed with the following priorities: to select the more reliable sequence in any given region, to extend sequence continuity by avoiding contig breaks, to minimize unnecessary switching between the hypercontigs, and to maximize the length of the reference sequence.

Before selecting the sequence for each reftig, we partitioned the hypercontig alignments into regions of high or low similarity. In regions of high similarity the only possible difference between the aligned sequences were single nucleotide polymorphisms, because gaps and contig breaks were by definition excluded from these regions. In these regions the reference sequence was selected base by base on the basis of read coverage, which we used as a proxy for sequence quality. Approximately half of the total alignment (containing about two-thirds of the bases in each haplome) was classified as highly similar. Highly similar regions had a mean length of 185 base pairs (bp) and an N50 of 330 bp.

A low similarity region was defined as the region flanked by two highly similar regions. Many of these regions contained haplome-specific sequence (polymorphic insertion/deletion events) and assembly gaps. As such, we were not always confident that the global alignment in these regions was entirely comprised of aligned allelic positions, because global aligners such as LAGAN are required to align each base and may therefore align nonhomologous bases. To avoid the creation of an artificial allele via an alignment artifact, the sequence of one hypercontig was selected for the entirety of each low similarity region. The selection was based on a set of heuristics designed to follow the priorities listed above (see Materials and methods, below). Low similarity regions accounted for approximately half of the total alignment, but contained only about one-third of the bases in each haplome. They had a mean length of 194 bp and an N50 of 2,675 bp.

### Reference sequence statistics

The *C. savignyi *reference sequence represents significant improvements in contiguity, continuity, and redundancy from the original WGS assembly (Table 3). The reference sequence has a total contig length of 174 Mb contained in 374 reftigs, of which the largest 100 contain 86% of the total sequence. The reftig N50 is 1.8 Mb and the contig N50 is 116 Kb, representing threefold and sevenfold improvements in contiguity over the original assembly (Table 3).

**Table 3:** Assembly statistics

	Reference sequence CSAV 2.0 (this work)	Original WGS assembly	Non-redundant 1.0
Total length (Mb)	174	402	157
Scaffold N50 (kb)	1,779	496	988
Contig N50 (kb)	116	17	47
Number of scaffolds	374	33,623	446
Number of scaffolds >3 kb	374	4,123	444
Number of contigs	4,620	66,800	8,183
Gap bases in scaffolds	1.7%	5.6%	4.3%

kb, kilobases; Mb, megabases; WGS, whole-genome shotgun.

The reference sequence also compares favorably with a previous nonredundant assembly that also used the original WGS assembly as a starting point ('nonredundant 1.0 assembly') [8]. This earlier assembly was generated by selecting a path through local alignments of the original WGS assembly with itself. Alignment discrepancies between the haplomes were resolved by breaking continuity rather than by resolution with assembly or genetic data. Compared with this earlier assembly, the reference sequence represents a twofold increase in scaffold and contig contiguity (Table 3). Additionally, the reference sequence is 10% longer (Table 3), and its largest 120 reftigs contain as many bases as all 446 supercontigs of the nonredundant 1.0 assembly.

In addition to extended contiguity, the continuity of the sequence has been improved in the reference sequence (Table 3). The frequency of gap bases ('N' placeholders whose number corresponds to the estimated size of the gap between adjacent contigs) has been decreased in the reference sequence to 1.7% of total positions, or 3 Mb. In comparison, 5.2% (22 Mb) of positions in the original WGS assembly and 4.3% (6.8 Mb) of positions in the nonredundant 1.0 assembly are gap bases. Increased continuity is also evident in the significant reduction in number of contigs, and hence decreased number of contig breaks (Table 3).

**Figure 6:**
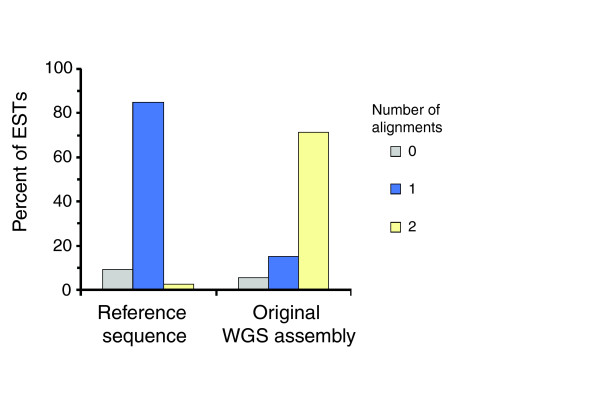
Redundancy is dramatically reduced in the reference sequence. Colored bars represent the percentage of *Ciona savignyi *expressed sequence tags (ESTs) aligning to each assembly a total of zero times (gray bar), exactly once (blue), and exactly twice (yellow). WGS, whole-genome shotgun.

The redundancy and completeness of the reference sequence were estimated by aligning the then available approximately 75,000 expressed sequence tags (ESTs) from *C. savignyi *to each assembly. Each EST was classified on whether it aligned to an assembly no, one, two, or more than two times. The EST alignments verify a significant reduction in redundancy in the reference sequence: 85% of ESTs align exactly once to the reference sequence whereas 72% align exactly twice to the original, redundant WGS assembly (Figure 6). By this same measure, the reference sequence is slightly less complete than the original WGS assembly, because 91% of ESTs align at least once to the reference sequence whereas 94% align at least once to the WGS original assembly. However, the reference sequence recovers 3% more ESTs than the nonredundant 1.0 assembly, to which 81% of ESTs align exactly once and 88% align at least once.

Of the reference sequence, 30% is classified as repetitive by RepeatMasker [36], utilizing the *de novo *RECON *C. savignyi *repeat library (see Materials and methods, below). By comparison, 38% of the original WGS assembly is classified as repetitive under the same conditions. This reduction in repeat content reflects the removal of uncondensed repetitive sequence fragments in the reference sequence pipeline. An annotated subset of the RECON library is available in the Repbase [37] database of mobile elements. Repeatmasker utilizing the annotated Repbase library classifies 16.7% of the reference sequence as mobile element derived and provides annotation of individual mobile element classes (Table 4). Short interspersed elements (SINEs) constitute the largest class of mobile element in the *C. savignyi *genome, accounting for 7.5% of bases in the reference sequence, followed by unclassified elements (3.4%), long interspersed elements (LINEs) (2.0%), DNA transposons (1.8%), and long terminal repeat (LTR) elements (1.3%).

**Table 4:** Mobile element content

	Total elements (haplome assembly)	Present in both haplomes ('ancestral')	Haplome-specific instances (insertions)	Ancestral/haplome specific
DNA transposons				
Charlie	6,286	4,484	684	6.6
En-Spm	101	51	10	5.1
Harbinger	403	184	61	3.0
hAT	4,111	1,783	872	2.0
Other	16,679	11,667	1,743	6.7
P	154	74	31	2.4
PiggyBac	22	14	5	2.8
Pogo	20	3	5	0.6
Tc2	746	517	68	7.6
Tip100	27	5	15	0.3
Retroelements				
SINEs	131,215	98,841	9,524	10.4
LINEs				
L1	4,468	2,203	552	4.0
L2	18,820	13,286	1,627	8.2
LOA	2,485	1,695	215	7.9
R2	526	298	87	3.4
RTE	162	109	22	5.0
LTR				
Gypsy	2,405	1,106	483	2.3
Pao	3,123	1,435	653	2.2
RC/Helitron	172	64	20	3.2
Unclassified	48,515	32,113	4,906	6.5
Satellites	8,301	5,417	738	7.3
Total	248,741	175,349	22,321	7.9

LINE, long interspersed element; LTR, long terminal repeat element; SINE, short interspersed element.

We did not detect anything unusual about the distribution of mobile elements in the reference sequence or between the aligned haplomes. The mobile element content of the two reconstructed haplomes is similar to that of the reference sequence, indicating that there was no detectable bias for or against annotated mobile element classes in the selection of the reference sequence. Overall, 248,741 Repbase mobile elements were identified in the dual haplome assembly. In total, 175,349 elements were present in the same alignment location as an annotation of the same element in the opposite haplome, and thus indicate an insertion event before the coalescence time of the two alleles. In all, 22,321 elements were aligned to alignment gaps in the opposite haplome, and therefore probably represent haplome-specific insertion events. The number of haplome-specific instances of mobile elements in each class is directly related to the total number of that element in the genome (Table 4). The remaining elements were unclassifiable because of missing sequence in the opposite haplome, fractured repeat annotation or alignment ambiguities. Detailed characterization of polymorphisms in the *C. savignyi *genome will be published elsewhere.

## Discussion

We constructed the nonredundant reference sequence of the *C. savignyi *genome from the initial, redundant WGS assembly. In this reference sequence, the vast majority of loci are represented exactly once. Compared with a previous nonredundant assembly [8], the contiguity of the sequence has been improved and identifiable misassemblies have been corrected. The reference sequence provides a valuable resource for both the *Ciona *research community and comparative genomics. It is the *C. savignyi *assembly currently available in Ensembl [38] and forms the basis of all currently available *C. savignyi *gene annotation sets [39]. We believe that the reference sequence is of high quality; as for all unfinished assemblies, however, users should anticipate the presence of some remaining misassemblies in the sequence. In particular, apparent duplications and copy number variation should be interpreted with caution because they could represent an undetected inclusion of both alleles of a polymorphic region. Additionally, because the reference sequence is a composite of the two haplomes of the sequenced individual, the sequence across a given region may not actually be present on the same haplotype in nature.

The *C. savignyi *reference sequence will facilitate comparative analysis, most importantly with the *C. intestinalis *genome. The two *Ciona *spp. are morphologically extremely similar and share nearly identical embryology [1]. *C. savignyi *and *C. intestinalis *hybrids are viable to the tadpole stage [40], but comparison of their genome sequence reveals a sequence divergence approximately equivalent to that seen between the human and chicken genomes. The combination of significant sequence divergence without significant functional divergence between these two species enables particularly powerful comparative sequence analysis [6,7]. To facilitate such comparisons, a whole-genome alignment of the *C. savignyi *reference sequence and v2.0 of the *C. intestinalis *assembly has been constructed and is available in the Vista genome browser [41,42] and through the Joint Genome Institute *C. intestinalis *genome browser [43]. Caution should be used in interpretation of species-specific duplications, which could be due to assembly artifacts.

A parallel goal of this work was to characterize polymorphism in the *C. savignyi *population. The high-quality, whole-genome alignment of the haplomes has facilitated identification of polymorphisms at multiple scales, including single nucleotide polymorphisms, insertion/deletion events and inversions, and sheds light on the population dynamics of highly polymorphic genomes [44].

The unusually deep raw sequence coverage accomplished by the *C. savignyi *genome sequencing project (>12×) allowed separate assembly of the two alleles, a critically important prerequisite for generating the reference sequence with the methodology we developed. This opportunity is unlikely to be reproduced in future genome assemblies. For example, when the recently completed Sea Urchin Genome Project was faced with a comparable level of heterozygosity within the single sequenced *Strongylocentrotus purpuratus *individual, they elected to adopt a hybrid approach, which combined 6× WGS sequencing data with 2× coverage of a bacterial artificial chromosome (BAC) minimal tiling path [21]. Because each BAC can only contain sequence from one of the two haplomes, the BAC sequence could then be used to separate allelic WGS reads during the assembly process. However, insights into misassemblies and the success of the general approach we described here should prove useful in informing assembly of other polymorphic species. We expect that as genome sequencing projects continue to move beyond inbred laboratory and agricultural strains, many more projects will be forced to adapt to the difficulties of polymorphic genome assembly. This has already been seen in the *C. intestinalis*, *Candida albicans*, *S. pupuratus*, *Anopheles *spp., and to a limited extent the fugu genome projects, and is anticipated to remain a significant problem as genome sequencing projects continue their rapid expansion.

## Conclusion

During the course of describing how we generated the nonredundant reference sequence of *C. savignyi*, we illustrated how the difficulties inherent in a WGS assembly of a highly polymorphic genome can be turned into an advantage with respect to the quality of the final sequence. The key step that facilitates this advantage is the alignment of the haplome assemblies, which allows correction of assembly errors that would go undetected in a standard WGS assembly, and dramatic extension of the continuity and contiguity of the reference sequence. The haplome alignment is dependent on the detection of allelic contigs, which in turn depends on having forced separate assembly of the two alleles during the course of producing the initial, redundant assembly. In the case of the *C. savignyi *genome, this strategy was possible because of the unprecedented depth to which its genome was sequenced. We believe that less than 12× coverage would be sufficient to pursue our strategy, but exactly where the cutoff would be is an area for further investigation. We also know that the extreme heterozygosity, which extended across the entire genome of the sequenced individual, facilitated the initial, separate assembly of the two alleles, but whether this strategy would work for less extremely polymorphic genomes is also an area for future work. We hope that the methodologic insights we generated will be as useful for future genome assemblies as the reference sequence will be for experimental work in *Ciona*.

## Materials and methods

### Assemblies

The original WGS assembly is available from the *Ciona savignyi *Database [45] at the Broad Institute. The reference sequence is available in Ensembl [37] and from the Sidow laboratory website [35].

### Repeat identification

Repeats were identified with RepeatMasker [36] utilizing a *de novo *repeat library constructed by the RECON [25] program and hand curated to remove multicopy genes, tRNA, and rRNA elements. The RECON library is available from the Sidow laboratory website [35].

### Resolution of spiders with a genetic cross

Fully informative genetic markers were designed at relevant locations surrounding each potential major misassembly and typed in 92 meioses of an outbred cross.

### Identifying unique anchors

The original WGS contig assembly was aligned to itself with a stringent optimization of WUBLAST [26]. If the stringent BLAST generated no hits of 100 bp with at least 95% identity or 200 bp with at least 90% identity, a second BLAST with less stringent parameters was executed. In practice, the majority of contigs without a hit in the first BLAST did not have a hit in the second BLAST either. Contig queries were soft-masked with Repeatmasker and the RECON library and the low complexity filter dust. The initial stringent BLASTN parameters were as follows: hitdist = 20, e cutoff = 1 × e^-20^, wink = 3, -topComboN = 1, and -Q (gap open penalty) = 40. In the second, less stringent BLAST the soft-masking parameters, topcomboN and e cutoff parameters were retained, and all other parameters were left at default.

Regions with exactly one hit to another contig for at least 100 consecutive base pairs were selected as 'anchors'. A total of 277,075 anchors connecting 33,684 contigs were identified. Of the 69,912 pieces evaluated (from a total of 66,799 contigs, because larger contigs were split into 30 kb pieces), 15,860 were completely masked or did not have an unmasked stretch of at least 11 bp on which WUBLAST could initiate a word hit. An additional 6,628 pieces did not have a single BLAST hit greater than 1 × e^-20 ^in either BLAST. Of the remaining 47,604 pieces, 36,404 (representing 33,684 contigs) were found to have at least one anchor and 11,200 did not contain a single anchor.

A potential source of error is an anchor between uniquely aligned masked regions. Masked regions were not included in the word generation stage of BLAST but were included in the alignment extension step. It is therefore possible that an entire anchor resides within a masked region adjacent to unmasked sequence that initiated a BLAST hit. To remove this possibility we screened all 277,075 anchors for anchors that did not contain at least 100 bp of consecutive unmasked bases. In all, 37,440 anchors (13.5% of all anchors, which connected 4,754 contigs) did not pass this test and were flagged and removed from later analysis. After the removal of the masked anchors, 239,635 anchors connecting 28,930 contigs remained.

### LAGAN alignment of anchored contigs

A global alignment was generated for all anchored contig pairs with the alignment program LAGAN [27] using default scoring parameters, except for the gap open penalty, which was decreased to -450. The alignments were rescored with the standard Smith-Waterman scoring of match = 5, mismatch = -4, gap open = -4, and gap extend per residue = -1, with the exception that terminal gaps were ignored and gap penalties were capped at 20 bp (corresponding to a score of -24). Gap penalties were capped to prevent overly penalizing aligned sequence adjacent to expected haplome-specific insertion/deletions events. LAGAN is known to produce a stereotypical error in which nonsimilar terminal regions are forced into alignment. To avoid this we ignored aligned end fragments that were less than 20 bp or less than 80% identical. Alignments with a score of less than 1,000 were considered spurious and eliminated from further analysis.

### Double Draft Aligner

The underlying idea behind the coassembly of two alleles is that each allele can be used to establish an ordering of the contigs and supercontigs in the other allele. Each allele is now a set of contigs (contiguous stretches of DNA sequence). The contigs are ordered into supercontigs by assembly links. These assembly links are based on paired reads, and are assumed to be less reliable than the assembled sequence in the contigs that they join [9]. To order allele A we use all contigs of allele B as ordering information, and then repeat the step to order allele B according to the contigs of allele A.

To accomplish this we used a sparse Dynamic Programming chaining algorithm [32]. This algorithm takes each contig of the base genome (or, in the case of *C. savignyi*, base haplome), and tiles it with local alignment from the second genome, taking into account not only sequence similarity but also common biologic rearrangement events, such as inversions and translocations. Because the two haplomes being co-assembled are very similar, we used a very high threshold for homology.

To order the contigs of allele A we did the following; for every pair of contigs from allele A (for instance, 1_A _and 2_A_) that are aligned next to each other in the tiled alignment of the contig X_B _(of allele B), we add a link joining 1_A _and 2_A_, and the two contigs are now said to be joined by an alignment link. The link is directed depending on the order and orientation of 1_A _and 2_A _hits on X_B_. If a contig has multiple forward or backward alignment links, it is labeled unreliable, because it could be a site of a misassembly on the contig level (or a biologic rearrangement). All links to unreliable contigs are removed. After this we use the assembly links that are not contradictory to the alignment links in order to increase the contiguity of the sequence. For any contig that is missing a forward or backward link but that has one in the original Arachne supercontig, we add this link to the link graph. After this, all connected components of the link graph are joined into a new haplome supercontig. The process is repeated in order to obtain a relative ordering of the connected components. During this step, only the reliable supercontigs of allele B are used as a basis for ordering all of the supercontigs of allele A and *vice versa*.

Note that during this procedure we may join with an alignment link two contigs that are in the same supercontig but that have other contigs in between them. Any such contig can be separated out into a new scaffold: if the in-between contigs match any sequence, then they will be aligned separately; and if they do not match any sequence, then we use the sequence from allele B to fill the sequence gap.

### Removal of tandem misassemblies

A purpose-built tool was designed to identify and remove tandemly misassembled alleles in adjacent contigs. The tool operates on an allelic 'bin' in which allelic supercontigs of a region have been collected and sorted into two sub-bins, corresponding to the two alleles of that region. Each contig was aligned with the local aligner CHAOS [46] to the preceding contig in its sub-bin, and all hits above a threshold of 5,000 (corresponding to about 50 aligned bases) were selected. The sequence of each selected hit was aligned with CHAOS to the entirety of both sub-bins to determine whether the sequence is unique to the adjacent contigs. If the sequence had no other instances in its own sub-bin and less than two hits on the opposite sub-bin, then it was considered a potential tandem misassembly. Real duplication events or repeated sequence motifs would be present in both sub-bins at a copy number of at least two and hence excluded at this stage. The tandemly misassembled region was removed from the preceding contig if the ratio of duplicated sequence to the length of nonduplicated sequence exceeded an empirical threshold. The tool was applied to adjacent contigs within supercontigs before the DDA step, and again on all adjacent contigs within hypercontigs after DDA. Because the contigs were repeat masked, tandemly misassembled repeat regions will not be identified.

### Hypercontig construction and alignment

Ordered contigs in each sub-bin were concatenated into a single hypercontig. A default gap of 10 'N's was inserted between all adjacent contigs without an Arachne gap estimate. Each pair of hypercontigs was aligned with LAGAN, using default scoring parameters with the exception of the gap open penalty, which was decreased to -450. Hypercontigs were masked with the full RECON library before alignment.

### Selecting the reference sequence

#### Annotation of high and low similarity regions

Before selection of the reference sequence the hypercontig alignments were partitioned into regions of high and low similarity. High similarity regions were identified by selecting aligned regions of perfect identity as seeds and expanding the seeds with a blast-like extension. The minimum seed length was 15 bp, the match score was set to 5, and the mismatch score to -4. If the cumulative score dropped below 95, or the extension encountered a supercontig break or a gapped alignment position, the extension was terminated and retracted to the last match. Low similarity regions were defined as the region between adjacent high similarity regions.

#### Annotation of sequence coverage

Read coverage was calculated for all positions in the original assembly by mapping read placement information from the Arachne output files onto contigs and counting the number of reads at each position. All hypercontig bases were mapped to their position in the original assembly and assigned the corresponding read coverage.

#### Selecting the reference sequence: regions of high similarity

In high similarity regions the reference sequence was selected at each position by comparing the read coverage of the aligned allelic bases and choosing the allele with read coverage closer to 6×, based on the assumption that bases with either low or extremely high read coverage are enriched for sequencing errors and assembly artifacts [9]. If the alleles had the same read coverage, the allele selected at the previous position was selected.

#### Selecting the reference sequence: regions of low similarity

In low similarity regions the sequence of one allele was selected for the entirety of the region based on the following heuristics.

If both alleles contained a contig break then the longer allele was selected.

If only one allele contained a contig break, then the unbroken allele was selected, unless the allele containing the contig break was longer than 20 kb and greater than 10 times the length of the continuous allele. This was done to avoid selecting against long regions that may have assembled in only one of the haplomes because of the draft nature of the assembly.

If neither allele contained a contig break the median read coverage of the bases in each allele was calculated. If both alleles did not have good median read coverage (3 ≤ X ≤ 15), then the allele with read coverage closer to the expected 6× coverage was selected. In a tie the longer allele was selected. If an allele was entirely gapped the read coverage of the previous position was used as a proxy. If both alleles had good median read coverage (3 ≤ X ≤ 15), then the repeat content of the region was examined. If the region was repetitive (90% of the longer allele was repeat masked) then the shorter allele was selected; otherwise the longer allele was selected.

### EST alignment

We used the same EST set and followed the same filtering procedure (removing about 250 ESTs of less than 100 bp and about 10,000 mitochondrial ESTs), as was employed in nonredundant assembly 1.0 [8]. We aligned the ESTs to each assembly with WUBLAST [26] and BLASTN in the place of BLAT [47], with the following parameters: -e = 10, -noseqs, -topcomboN = 1, -links, and -Q = 20. As in the report by Vinson and coworkers [8], all alignments in which matching bases exceeded 80% of the length of the EST were retained. Our WUBLAST yielded virtually the same number of alignments in all categories as the BLAT analysis [8].

## Additional data files

The following data are available with the online version of this paper. Additional data file 1 is a figure of a representative alignment 'spider' involving sequence from three bins. Additional data file 2 is a figure displaying heavy enrichment for low coverage bases in unassigned sequence. Additional data file 3 is a figure displaying the length distribution of predicted contig overlaps in the original WGS assembly.

## Supplementary Material

Additional data file 1 In this figure supercontigs are black lines denoted with purple letters. Regions of long alignment are denoted in turquoise (approximately to scale), with the length of the alignment in megabases. Regions of short alignment are denoted in pink (grossly out of scale to make them visible), with the length of the alignment in kilobases. Approximate positions of genetic markers are given by orange ovals, and their names are in blue. Linkage is shown by a red dashed line with the genetic distance indicated. Lack of a red dashed line between any two markers indicates no detectable linkage. Positions where the assembly is broken to account for the genetic map data are shown as circled lightning. Click here for file

Additional data file 2 In this figure the x-axis diplays the maximum read coverage per contig and the y-axis displays the percentage of contigs in a category. Red bars indicate unassigned contigs, and blue bars indicate contigs assigned to an allelic bin. Click here for file

Additional data file 3 A figure displaying the length distribution of predicted contig overlaps in the original WGS assembly. Click here for file

## References

[B1] Satoh N The ascidian tadpole larva: comparative molecular development and genomics. Nat Rev Genet 2003 4 285 295 12671659 10.1038/nrg1042

[B2] Di Gregorio A Levine M Analyzing gene regulation in ascidian embryos: new tools for new perspectives. Differentiation 2002 70 132 139 12147132 10.1046/j.1432-0436.2002.700402.x

[B3] Satoh N Developmental Biology of Ascidians 1994 Cambridge: Cambridge University Press

[B4] Shi W Levine M Davidson B Unraveling genomic regulatory networks in the simple chordate, *Ciona intestinalis*. Genome Res 2005 15 1668 1674 16339364 10.1101/gr.3768905

[B5] Dehal P Satou Y Campbell RK Chapman J Degnan B De Tomaso A Davidson B Di Gregorio A Gelpke M Goodstein DM The draft genome of *Ciona intestinalis*: insights into chordate and vertebrate origins. Science 2002 298 2157 2167 12481130 10.1126/science.1080049

[B6] Bertrand V Hudson C Caillol D Popovici C Lemaire P Neural tissue in ascidian embryos is induced by FGF9/16/20, acting via a combination of maternal GATA and Ets transcription factors. Cell 2003 115 615 627 14651852 10.1016/s0092-8674(03)00928-0

[B7] Johnson DS Davidson B Brown CD Smith WC Sidow A Noncoding regulatory sequences of *Ciona *exhibit strong correspondence between evolutionary constraint and functional importance. Genome Res 2004 14 2448 2456 15545496 10.1101/gr.2964504 PMC534669

[B8] Vinson JP Jaffe DB O'Neill K Karlsson EK Stange-Thomann N Anderson S Mesirov JP Satoh N Satou Y Nusbaum C Assembly of polymorphic genomes: algorithms and application to *Ciona savignyi*. Genome Res 2005 15 1127 1135 16077012 10.1101/gr.3722605 PMC1182225

[B9] Batzoglou S Jaffe DB Stanley K Butler J Gnerre S Mauceli E Berger B Mesirov JP Lander ES ARACHNE: a whole-genome shotgun assembler. Genome Res 2002 12 177 189 11779843 10.1101/gr.208902 PMC155255

[B10] Jaffe DB Butler J Gnerre S Mauceli E Lindblad-Toh K Mesirov JP Zody MC Lander ES Whole-genome sequence assembly for mammalian genomes: Arachne 2. Genome Res 2003 13 91 96 12529310 10.1101/gr.828403 PMC430950

[B11] Huang X Wang J Aluru S Yang SP Hillier L PCAP: a whole-genome assembly program. Genome Res 2003 13 2164 2170 12952883 10.1101/gr.1390403 PMC403719

[B12] Myers EW Sutton GG Delcher AL Dew IM Fasulo DP Flanigan MJ Kravitz SA Mobarry CM Reinert KH Remington KA A whole-genome assembly of *Drosophila*. Science 2000 287 2196 2204 10731133 10.1126/science.287.5461.2196

[B13] Mullikin JC Ning Z The phusion assembler. Genome Res 2003 13 81 90 12529309 10.1101/gr.731003 PMC430959

[B14] Venter JC Adams MD Myers EW Li PW Mural RJ Sutton GG Smith HO Yandell M Evans CA Holt RA The sequence of the human genome. Science 2001 291 1304 1351 11181995 10.1126/science.1058040

[B15] Waterston RH Lindblad-Toh K Birney E Rogers J Abril JF Agarwal P Agarwala R Ainscough R Alexandersson M An P Initial sequencing and comparative analysis of the mouse genome. Nature 2002 420 520 562 12466850 10.1038/nature01262

[B16] Adams MD Celniker SE Holt RA Evans CA Gocayne JD Amanatides PG Scherer SE Li PW Hoskins RA Galle RF The genome sequence of *Drosophila melanogaster*. Science 2000 287 2185 2195 10731132 10.1126/science.287.5461.2185

[B17] Hillier LW Miller W Birney E Warren W Hardison RC Ponting CP Bork P Burt DW Groenen MA Delany ME Sequence and comparative analysis of the chicken genome provide unique perspectives on vertebrate evolution. Nature 2004 432 695 716 15592404 10.1038/nature03154

[B18] Jones T Federspiel NA Chibana H Dungan J Kalman S Magee BB Newport G Thorstenson YR Agabian N Magee PT The diploid genome sequence of *Candida albicans*. Proc Natl Acad Sci USA 2004 101 7329 7334 15123810 10.1073/pnas.0401648101 PMC409918

[B19] Holt RA Subramanian GM Halpern A Sutton GG Charlab R Nusskern DR Wincker P Clark AG Ribeiro JM Wides R The genome sequence of the malaria mosquito *Anopheles gambiae*. Science 2002 298 129 149 12364791 10.1126/science.1076181

[B20] Aparicio S Chapman J Stupka E Putnam N Chia JM Dehal P Christoffels A Rash S Hoon S Smit A Whole-genome shotgun assembly and analysis of the genome of *Fugu rubripes*. Science 2002 297 1301 1310 12142439 10.1126/science.1072104

[B21] Sodergren E Weinstock GM Davidson EH Cameron RA Gibbs RA Angerer RC Angerer LM Arnone MI Burgess DR Burke RD The genome of the sea urchin *Strongylocentrotus purpuratus*. Science 2006 314 941 952 17095691 10.1126/science.1133609 PMC3159423

[B22] Brent MR Genome annotation past, present, and future: how to define an ORF at each locus. Genome Res 2005 15 1777 1786 16339376 10.1101/gr.3866105

[B23] Korf I Gene finding in novel genomes. BMC Bioinformatics 2004 5 59 15144565 10.1186/1471-2105-5-59 PMC421630

[B24] Curwen V Eyras E Andrews TD Clarke L Mongin E Searle SM Clamp M The Ensembl automatic gene annotation system. Genome Res 2004 14 942 950 15123590 10.1101/gr.1858004 PMC479124

[B25] Bao Z Eddy SR Automated de novo identification of repeat sequence families in sequenced genomes. Genome Res 2002 12 1269 1276 12176934 10.1101/gr.88502 PMC186642

[B26] WU-BLAST http://blast.wustl.edu

[B27] Brudno M Do CB Cooper GM Kim MF Davydov E Green ED Sidow A Batzoglou S LAGAN and Multi-LAGAN: efficient tools for large-scale multiple alignment of genomic DNA. Genome Res 2003 13 721 731 12654723 10.1101/gr.926603 PMC430158

[B28] Salzberg SL Yorke JA Beware of mis-assembled genomes. Bioinformatics 2005 21 4320 4321 16332717 10.1093/bioinformatics/bti769

[B29] Celniker SE Wheeler DA Kronmiller B Carlson JW Halpern A Patel S Adams M Champe M Dugan SP Frise E Finishing a whole-genome shotgun: release 3 of the *Drosophila melanogaster *euchromatic genome sequence. Genome Biol 2002 3 RESEARCH0079 12537568 10.1186/gb-2002-3-12-research0079 PMC151181

[B30] Warren RL Varabei D Platt D Huang X Messina D Yang SP Kronstad JW Krzywinski M Warren WC Wallis JW Physical map-assisted whole-genome shotgun sequence assemblies. Genome Res 2006 16 768 775 16741162 10.1101/gr.5090606 PMC1473187

[B31] Semple CA Morris SW Porteous DJ Evans KL Computational comparison of human genomic sequence assemblies for a region of chromosome 4. Genome Res 2002 12 424 429 11875030 10.1101/gr.207902 PMC155292

[B32] Sundararajan M Brudno M Small KS Sidow A Batzoglou S Chaining algorithms for alignment of draft sequence. Proceedings of the Fourth Workshop on Algorithms in Bioinformatics (WABI 2004); 17-21 September 2004 2004 Heidelberg, Germany: Springer-Verlag

[B33] Brudno M Malde S Poliakov A Do CB Couronne O Dubchak I Batzoglou S Glocal alignment: finding rearrangements during alignment. Bioinformatics 2003 Suppl 1 i54 i62 10.1093/bioinformatics/btg1005 12855437

[B34] Pop M Kosack DS Salzberg SL Hierarchical scaffolding with Bambus. Genome Res 2004 14 149 159 14707177 10.1101/gr.1536204 PMC314292

[B35] The *Ciona Savignyi *Reference Genome http://mendel.stanford.edu/sidowlab/ciona.html

[B36] RepeatMasker Open-3.0 http://www.repeatmasker.org

[B37] Jurka J Kapitonov VV Pavlicek A Klonowski P Kohany O Walichiewicz J Repbase Update, a database of eukaryotic repetitive elements. Cytogenet Genome Res 2005 110 462 467 16093699 10.1159/000084979

[B38] Birney E Andrews D Caccamo M Chen Y Clarke L Coates G Cox T Cunningham F Curwen V Cutts T Ensembl 2006. Nucleic Acids Res 2006 34 D556 D561 16381931 10.1093/nar/gkj133 PMC1347495

[B39] Ensembl *Ciona savignyi *genome browser http://www.ensembl.org/Ciona_savignyi

[B40] Byrd J Lambert CC Mechanism of the block to hybridization and selfing between the sympatric ascidians *Ciona intestinalis *and *Ciona savignyi*. Mol Reprod Dev 2000 55 109 116 10602281 10.1002/(SICI)1098-2795(200001)55:1<109::AID-MRD15>3.0.CO;2-B

[B41] Frazer KA Pachter L Poliakov A Rubin EM Dubchak I VISTA: computational tools for comparative genomics. Nucleic Acids Res 2004 32 W273 W279 15215394 10.1093/nar/gkh458 PMC441596

[B42] The VISTA genome browser http://pipeline.lbl.gov

[B43] The *Ciona intestinalis *genome browser http://genome.jgi-psf.org/Cioin2/Cioin2.home.html

[B44] Small K Brudno M Hill M Sidow A Extreme genomic variation in a natural population. Proc Natl Acad Sci USA 2007 in press 10.1073/pnas.0700890104 PMC1838466 17372217

[B45] The *Ciona savignyi *Database http://www.broad.mit.edu/annotation/ciona/

[B46] Brudno M Morgenstern B Fast and sensitive alignment of large genomic sequences. Proc IEEE Comput Soc Bioinform Conf 2002 1 138 147 15838131

[B47] Kent WJ BLAT: the BLAST-like alignment tool. Genome Res 2002 12 656 664 11932250 10.1101/gr.229202 PMC187518

